# Central auditory processing and self-perception questionnaire after acoustically controlled auditory training in individuals with mild traumatic brain injury

**DOI:** 10.1590/2317-1782/20232023048en

**Published:** 2024-04-26

**Authors:** Ana Karina Lima Buriti, Cyntia Barbosa Laureano Luiz, Laís Rocha de Barros Oliveira, Italo Capraro Suriano, Daniela Gil

**Affiliations:** 1 Departamento de Fonoaudiologia, Universidade Federal de São Paulo - UNIFESP - São Paulo (SP), Brasil.

**Keywords:** Hearing, Speech Perception, Auditory Perception, Cognition, Brain Injury, Quality of Life

## Abstract

**Purpose:**

To correlate behavioral assessment results of central auditory processing and the self-perception questionnaire after acoustically controlled auditory training.

**Methods:**

The study assessed 10 individuals with a mean age of 44.5 years who had suffered mild traumatic brain injury. They underwent behavioral assessment of central auditory processing and answered the Formal Auditory Training self-perception questionnaire after the therapeutic intervention - whose questions address auditory perception, understanding orders, request to repeat statements, occurrence of misunderstandings, attention span, auditory performance in noisy environments, telephone communication, and self-esteem. Patients were asked to indicate the frequency with which the listed behaviors occurred.

**Results:**

Figure-ground, sequential memory for sounds, and temporal processing correlated with improvement in following instructions, fewer requests to repeat statements, increased attention span, improved communication, and understanding on the phone and when watching TV.

**Conclusion:**

Auditory closure, figure-ground, and temporal processing had improved in the assessment after the acoustically controlled auditory training, and there were fewer auditory behavior complaints.

## INTRODUCTION

Traumatic brain injury (TBI) is caused by a blow or violent shock to the skull, with consequences on the brain. TBI severity is classified by the level of consciousness at the time of hospital admission after the accident, using the Glasgow Coma Scale. It evaluates the patient's eye opening and verbal and motor response, assigning a score ranging from 3 to 15. A TBI is considered mild when this score is between 13 and 15; moderate when it is between 9 and 12; and severe when it is between 3 and 8^(1)^.

Central auditory processing (CAP) assessment is known to be important in patients who have suffered TBI. However, those with mild injuries may have symptoms such as reduced information processing speed and deficits in attention, orientation, executive function, and language in the long run, which may have an impact on their ability to process auditory information^(2)^.

An American study reported CAP disorder in more than 55% of adults and children who suffered TBI. The authors recommend that patients with TBI be referred to therapy to help improve auditory skills through auditory training, aiming to compensate for residual losses, using central top-down resources (from the nervous system to the periphery) - i.e., with metacognitive and cognitive strategies and metalinguistic skills^(3)^.

International recommendations have highlighted the importance of auditory training strategies and exercises to promote efficient perceptions and minimize functional listening deficits through cortical reorganization and plasticity in individuals with CAP disorder. They have also referred to each person’s specificities, especially those with comorbidities (such as TBI), advising against either overestimating or underestimating their cognitive, language, and intellectual capacity, as it could compromise their motivation, which is essential to therapy^(4)^.

Werff^(2)^ has stated that TBI is unlikely to damage only specific central hearing centers. However, central auditory manifestations in mild TBI must be assessed, which requires speech-language-hearing therapy planning to induce neurophysiological changes and improve auditory skills. The author mentioned the lack of a standardized battery of tests. However, it should include at least speech-in-noise, temporal resolution, and dichotic listening tests with binaural separation or selective attention tasks.

Changing the environment is one of the valid guidelines for individuals with mild TBI, as it makes sound clearer and more accessible, improves signal-to-noise ratio, and increases the ability to hear and learn from auditory signals. Such changes can and should be related to cognitive training strategies^(2)^ used by multidisciplinary teams, including a sound-booth auditory training approach known as acoustically controlled auditory training (ACAT).

International guidelines have already recommended that health professionals or educators select children or adults with possible risk factors for CAP disorder, using inventories or questionnaires that identify possible changes related to listening comprehension, academic issues, and social and workplace skills. On the other hand, although questionnaires help professionals identify complaints and map difficulties, they do not replace the complete assessment indicated for diagnosing CAP disorder^(5)^.

The national literature indicates many hearing self-perception questionnaires, but it lacks research analyzing individuals with comorbidities after information processing diagnosis and therapeutic intervention, specifically involving mild TBI, which reveals a gap in the literature^(6-7)^.

Hence, it is relevant to investigate the impact of mild TBI on the ability to process auditory information and the person's self-perception after a therapeutic approach. This study contributes to the possibility of validating the benefits of the therapeutic approach in question from the patient's perspective.

Therefore, this study aimed to correlate behavioral CAP assessment results with a self-perception questionnaire after ACAT in individuals with mild TBI.

## METHODS

This quantitative descriptive research was carried out at the clinical audiology outpatient clinic of the course on Hearing Disorders at the Speech-Language-Hearing Department of the Federal University of São Paulo (UNIFESP) and approved by the Research Ethics Committee at UNIFESP under number 1.844.535.

The sample comprised 10 individuals (two females and eight males), aged 16 to 64 years, with a medical diagnosis of mild closed TBI.

The sample was selected and recruited at the neurotrauma and neurosurgery outpatient clinic at the São Paulo Hospital. Individuals who agreed to participate in the study signed an informed consent form and were invited to an initial assessment session to ensure they met the study's inclusion criteria, namely: absence of previous or current complaints of auditory system disorders; having suffered a TBI between 4 and 12 months before; hearing thresholds less than or equal to 25 dBHL between 250 and 4000 Hz, bilaterally; type-A tympanometry; and absence of diagnosed and/or evident behavioral or psychiatric changes.

Then, their CAP was submitted to behavioral assessment with the following 10 tests: sound localization test, sequential memory test for verbal and nonverbal sounds, speech-in-noise, sentence identification with an ipsilateral competing message, dichotic staggered spondaic word, pure-tone duration pattern^(8)^, dichotic consonant-vowel, Random Gap Detection Test, and Masking-Level Difference. The tests used a Grason-Stadler audiometer, model GSI-61, with TDH-50P earphones.

After this assessment, individuals were invited to attend 10 ACAT sessions, following Dias and Gil´s protocol^(9)^, with 50-minute weekly sessions in a sound booth.

The sessions included increasingly complex activities to train and stimulate the target auditory skills, using stimuli recorded on a CD and presented through earphones via an audiometer in progressively adverse dichotic, monotic, or diotic listening conditions - i.e., from the easiest to the most difficult level.

The ACAT program in this study lasted an average of 3 months for each patient, including absences and holidays. The proposed activities were the same for all study participants.

The activities in the ACAT sessions involved the following skills: verbal and nonverbal sound recognition and discrimination, temporal ordering, temporal resolution, figure-ground for verbal and non-verbal sounds, and auditory closure (Chart 1). The tasks and the signal-to-noise ratios progressed with the level of complexity. Participants had to achieve at least 70% correct answers to move on to the subsequent auditory training stage^(10)^.

**Chart 1:** Timeline of acoustically controlled auditory training activities

1^st^ SESSION	Figure-ground for sentences.
2^nd^ SESSION	Figure-ground for words: targeted listening with digits RE + figure-ground for nonverbal sounds LE.
3^rd^ SESSION	Figure-ground for words: targeted listening with digits LE + figure-ground for nonverbal sounds RE.
4^th^ SESSION	Binaural integration with digits and nonverbal sounds + speech in noise with sentences.
5^th^ SESSION	Auditory closure (speech in noise: sentences, figures, and words) RE and LE.
6^th^ SESSION	Temporal aspects (intensity pattern) + temporal aspects (duration pattern: audiometer and flute).
7^th^ SESSION	Temporal aspects (duration pattern: pure tone).
8^th^ SESSION	Temporal aspects (frequency pattern: audiometer, pure tone).
9^th^ SESSION	Temporal aspects (frequency pattern: flute + figure-ground for syllables (RE).
10^th^ SESSION	Figure-ground for syllables (LE) and binaural integration with syllables.
Reassessment	CAP reassessment + self-perception questionnaire.

**Caption:** RE: right ear; LE: left ear; CAP: central auditory processing

At the end of the 10 ACAT sessions, they were reassessed using the same initial protocol. All pre-training and post-training tests were analyzed according to Pereira’s criteria^(11,12)^.

Then, they answered the Post-Formal Auditory Training self-perception questionnaire, translated and adapted into Brazilian Portuguese by Dias and Gil^(9)^, used in individuals with and without hearing loss to formally score the changes brought about by auditory training from the person’s or their family’s perspective. It has 12 questions on the perception of hearing improvement, understanding orders, academic progress, requests to repeat statements, fewer misunderstandings, increased attention span, auditory performance in noisy environments, improvements when talking on the phone or watching television, and self-esteem. They were instructed to assign their self-perceived response to each item on a scale ranging from 0 to 4, in which 0 meant no improvement; 1, subtle but important improvement; 2, moderate improvement; 3, considerable improvement; and 4, significant improvement (Chart 2). The researcher gave an example in percentages ranging from 0 to 100% improvement to help them understand the scale and clearly answer each question.

**Chart 2:** Self-perception questionnaire (after acoustically controlled auditory training)

**Response analysis:** 0- no improvement; 1- subtle but important improvement; 2- moderate improvement; 3- considerable improvement; 4- significant improvement.	0	1	2	3	4
Q1. Have you noticed any improvement in your hearing?					
Q2. Have you noticed any improvement in following instructions?					
Q3. Has communication become easier?					
Q4. Has there been any academic improvement (reading, spelling?)					
Q5. Have you been asking to have statements repeated less often?					
Q6. Have there been fewer communication misunderstandings?					
Q7. Has your attention span increased?					
Q8. Has your hearing performance in noisy environments improved?					
Q9. Have your attention and alertness levels improved?					
Q10. Have you noticed any improvement in speaking on the phone, watching TV, listening to the radio, etc.?					
Q11. Has your self-esteem improved?					
Q12. Please, describe other changes perceived during or after the formal auditory training.					

The data were descriptively analyzed based on absolute and percentage frequencies for discrete variables and measurements of mean, standard deviation (mean ± SD), and median for numerical variables. The Student's and Wilcoxon’s paired t-tests were used for inferential statistical analysis. The margin of error used in the decision of statistical tests was p < 0.05%.

## RESULTS

The sample’s ages ranged from 16 to 64 years, with a mean of 44.50 years, standard deviation of 18.32 years, and median of 53.00 years. Most patients were males (80%). They had the following causes for TBI: falling from a height greater than 2 meters (40%), falling from their own height (30%), and car accidents (30%).

The TBI of the individuals in this study affected mostly the left side (40%), followed by both sides (40%) and the right side (20%).

Table 1 shows the characterization of the sample regarding their age, sex, medical diagnosis of the injury, whether primary or secondary to mild TBI, scoring 13 to 15 in Glasgow at the time of admission to the hospital, the side of the injury, whether they underwent a surgical procedure, and medication use.

**Table 1:** Characterization of the sample

**N**	**Age**	**Sex**	**Education level**	**Medical diagnosis**	**Injury side**	**Surgery**	**Length of hospital stay**	**Medication**
1	51	M	High school, incomplete	Chronic subdural hematoma	Left	Yes	3 days	No
2	64	M	High school diploma	Frontal and parietal intraparenchymal hematoma	Left	No	3 days	Sertraline
3	58	M	Middle school, incomplete	Acute subdural hematoma	Left	No	4 days	No
4	32	F	Middle school, incomplete	medial frontal contusion + temporal contusion	Bilateral	No	1 day	No
5	16	M	High school, incomplete	Frontal extradural empyema	Left	Yes	2 months 3 ICU.	No
6	64	F	Bachelor’s degree	Frontal contusion	Right	No	3 days	Sertraline
7	28	M	High school diploma	Parietal extradural hematoma	Right	Yes	4 days	No
8	21	M	High school diploma	Diffuse concussion	Bilateral	No	No	Fluoxetine
9	55	M	Middle school, incomplete	Temporal extradural hematoma + Acute epidural hematoma + Acute laminar frontotemporal subdural hematoma	Bilateral	Yes	5 days	No
10	56	M	High school diploma	Chronic subdural hematoma	Bilateral	Yes	3 days	No

**Caption:** M: males; F: females; ICU: intensive care unit

The behavioral CAP test results before and after ACAT are shown in Table 2.

**Table 2:** Behavioral assessment of the central auditory processing before and after acoustically controlled auditory training per test and ear (n = 10)

		**Assessment**		
**Behavioral tests**	**Ear**	**Before**	**After**	**p-value**
		Mean ± SD (Median)	Mean ± SD (Median)	
				
**SWNT** (%)	Right	67.60 ± 16.91 (70.00)	76.80 ± 10.29 (80.00)	p^(1)^ = 0.127
	Left	65.60 ± 7.11 (64.00)	74.40 ± 10.36 (74.00)	p^(2)^ = 0.042*
	**p-value**	p^(1)^ = 0.678	p^(1)^ = 0.329	
				
**SIICM (SNR -15)** (%)	Right	63.00 ± 17.67 (65.00)	75.00 ± 15.09 (70.00)	p^(1)^ = 0.074
	Left	61.00 ± 17.29 (70.00)	71.00 ± 18.53 (80.00)	p^(2)^ = 0.093
	**p-value**	p^(1)^ = 0.678	p^(1)^ = 0.399	
				
**SSW** (%)	Right	83.00 ± 17.11 (88.75)	89.75 ± 12.44 (93.75)	p^(2)^ = 0.016*
	Left	81.75 ± 13.02 (85.00)	85.00 ± 16.41 (90.00)	p^(2)^ = 0.108
	**p-value**	p^(2)^ = 0.435	p^(1)^ = 0.038*	
				
**DCVT** (correct answers)	Right	10.40 ± 3.44 (10.50)	12.10 ± 2.96 (11.50)	p^(1)^ = 0.169
	Left	8.00 ± 3.20 (7.50)	6.50 ± 2.55 (6.50)	p^(1)^ = 0.169
	**p-value**	p^(1)^ = 0.217	p^(1)^ = 0.009*	
				
**DCVT** (wrong answers)		5.60 ± 3.37 (4.00)	5.20 ± 1.40 (5.00)	p^(1)^ = 0.674
				
**SLT** (%)		68.00 ± 19.32 (80.00)	82.00 ± 19.89 (80.00)	p^(2)^ = 0.020*
				
**SMTV** (%)		50.00 ± 36.00 (50.00)	73.33 ± 21.08 (66.66)	p^(^1^)^ = 0.089
				
**SMTNV** (%)		63.33 ± 36.68 (66.66)	73.33 ± 34.43 (83.33)	p^(^2^)^ = 0.257
				
**RGDT** (ms)		10.25 ± 6.67 (8.13)	5.60 ± 2.77 (4.50)	p^(1)^ = 0.036*
				
**DPT** (%)		62.53 ± 26.85 (64.97)	79.32 ± 24.40 (88.33)	p^(1)^ = 0.133
				
**MLD** (dB)		12.80 ± 3.43 (12.00)	12.20 ± 2.57 (12.00)	p^(1)^ = 0.691
				

* Significant difference at 5.0%

^(1)^with Student’s paired t-test;

^(2)^with Wilcoxon’s paired t-test

**Caption:** SWNT: speech-in-white-noise test; SIICM: sentence identification with ipsilateral competing message; SSW: dichotic staggered spondaic word; DCVT: dichotic consonant-vowel test; SLT: sound localization test; SMTV: sequential memory test for verbal sounds; SMTNV: sequential memory test for nonverbal sounds; DPT: duration pattern test; RGDT: Random Gap Detection Test; MLD: masking-level difference

A statistically significant difference was found between before and after ACAT in the speech-in-noise test in the left ear (p = 0.042), in the dichotic staggered spondaic word test in the right ear (p = 0.016), in the sound localization test (p = 0.020), and in the Random Gap Detection Test (p = 0.036), with better performances in the reassessment - i.e., after ACAT. A statistically significant difference was also observed between the right and left ears in the reassessment with the dichotic staggered spondaic word test (p = 0.038) and dichotic consonant-vowel test (p = 0.009) (Table 2).

As for hemispheric dominance, the right had an advantage in the dichotic consonant-vowel test before and after ACAT.

Table 3 presents the participants' responses regarding self-perceived information-processing behaviors after ACAT.

**Table 3:** Frequency distribution of responses to the Formal Auditory Training Questionnaire

	**Improvement response levels**									
**FAT**	**None**		**Subtle**		**Moderate**		**Considerable**		**Significant**	
	n	%^(1)^	n	%^(1)^	n	%^(^1^)^	n	%^(1)^	n	%^(1)^
										
**Q1**	-	-	-	-	1	10.0	5	50.0	4	40.0
										
**Q2**	1	10.0	-	-	2	20.0	4	40.0	3	30.0
										
**Q3**	1	10.0	1	10.0	1	10.0	4	40.0	3	30.0
										
**Q4**	2	20.0	2	20.0	2	20.0	1	10.0	3	30.0
										
**Q5**	-	-	1	10.0	3	30.0	3	30.0	3	30.0
										
**Q6**	1	10.0	-	-	2	20.0	4	40.0	3	30.0
										
**Q7**	-	-	1	10.0	1	10.0	3	30.0	5	50.0
										
**Q8**	2	20.0	-	-	1	10.0	1	10.0	6	60.0
										
**Q9**	1	10.0	-	-	2	20.0	1	10.0	6	60.0
										
**Q10**	1	10.0	1	10.0	-	-	5	50.0	3	30.0
										
**Q11**	1	10.0	1	10.0	-	-	2	20.0	6	60.0
										

^(1)^Percentages were obtained from all 10 patients analyzed

**Caption:** FAT: formal auditory training; Q: question; Q1: Have you noticed any improvement in your hearing?; Q2: Have you noticed any improvement in following instructions and orders?; Q3: Has communication become easier?; Q4: Has there been any academic improvement (reading, spelling?); Q5: Have you been asking to have statements repeated less often?; Q6: Have there been fewer communication misunderstandings?; Q7: Has your attention span increased?; Q8: Has your hearing performance in noisy environments improved?; Q9: Have your attention and alertness levels improved?; Q10: Have you noticed any improvement in speaking on the phone, watching TV, listening to the radio, etc.?; Q11: Has your self-esteem improved?

Table 3 shows a greater concentration of responses in the columns of considerable and significant improvement, highlighting questions Q8 (hearing performance in noisy environments), Q9 (level of attention and alertness) and Q11 (how much self-esteem) in the column of significant improvement and Q1 (improvement in hearing) and Q10 (when talking on the phone, watching TV, listening to the radio) in that of considerable improvement, followed by Q2, Q3, and Q6.

The statistically significant results of the Formal Auditory Training questionnaire responses are shown in Table 4, correlated with the behavioral information test results after ACAT.

**Table 4:** Correlation between the Formal Auditory Training questionnaire and behavioral tests after acoustically controlled auditory training

	**FAT**					
**CAP - After**	**Q2**	**Q5**	**Q7**	**Q8**	**Q9**	**Q10**
**SWNT**						
Right	-	-	-	-	-0.763 (0.010)^(2)*^	-
**SIICM**						
Left	0.911 (< 0.001)^(2)*^	-	-	-	-	-
**DCVT**						
Right	-	0.734 (0.016)^(^1)^*^	-	-	-	-
**SMTV**	-	-	-	-0.639 (0.047)^(2)*^	-	-
**SMTNV**	0.633 (0.050)^(^2)^*^	-	-	-	-	-
**RGDT**	-0.823 (0.003)^(2)*^	-	-	-	-	-
**DPT**	0.779 (0.008)^(2)*^	-	0.710 (0.021)^(2)^*	-	-	0.684 (0.029)^(2)*^

* Statistically different from zero

^(1)^Pearson correlation;

^(2)^Spearman correlation

**Caption:** CAP: central auditory processing; FAT: formal auditory training; Q2: Have you noticed any improvement in following instructions and orders?; Q5: Have you been asking to have statements repeated less often?; Q7: Has your attention span increased?; Q8: Has your hearing performance in noisy environments improved?; Q9: Have your attention and alertness levels improved?; Q10: Have you noticed any improvement in speaking on the phone, watching TV, listening to the radio, etc.?; SWNT: speech-in-white-noise test; SIICM: sentence identification with an ipsilateral competing message; DCVT: dichotic consonant-vowel test; SMTV: sequential memory test for verbal sounds; SMTNV: sequential memory test for nonverbal sounds; RGDT: Random Gap Detection Test; DPT: duration pattern test

Post-ACAT behavioral tests were positively correlated with the post-ACAT questionnaire, as patients who reported improvements in following instructions and orders (Q2) also had better results in sentence identification with an ipsilateral competing message (p < 0.001) in the left ear, sequential memory test for nonverbal sounds (0.050), Random Gap Detection Test (0.003), and duration pattern test (p = 0.008).

Participants reported a decrease in requests to repeat statements (Q5) with significance in the dichotic vowel-consonant test and an advantage in the right ear (p = 0.016). Regarding attention difficulties, the patients reported an increase in their attention span (Q7) and improvements in talking on the phone, watching TV, and listening to the radio (Q10), with a statistically significant difference only in the duration pattern test (respectively, p = 0.021 and p = 0.029) (Table 4). This correlation was therefore positive, in that the greater the perceived improvement in attention span, the better the performance in temporal aspects in identifying sound duration. It can be inferred that by being able to remain more attentive, the individual improved their ability to distinguish sound duration, impacting communicative exchanges with a better use of the supra-segmental content of speech (tone and intonation).

The hearing performance in noisy environments (Q8) and the level of attention and alertness (Q9) were statistically different in the sequential memory test for verbal sounds (p = 0.047) and speech-in-white-noise test (p = 0.010), respectively. This correlation was negative, which makes it possible to state that the worse the hearing performance in a noisy environment, the better the performance in memorizing verbal sounds. Also, the worse the hearing performance in a noisy environment, the better the level of attention and alertness.

## DISCUSSION

Mild TBI increases diffusion in cortical gray matter, and neurobehavioral and physiological signs decrease 4 months after the injury. Authors^(13)^ reported that the individual may not have changes in the cortical or subcortical region, although mild TBI patients may have late neuronal loss. Hence, they may have auditory symptoms, which makes it important to carry out peripheral and central audiological assessments 4 months after the TBI, when many other clinical symptoms stabilize.

The descriptive analysis of the behavioral CAP assessment (Table 2) found a statistically significant difference between mean pre- and post-ACAT correct answers in the speech-in-noise tests in the left ear, staggered spondaic word in the right ear, sound localization test, and Random Gap Detection Test. It demonstrates that auditory training improved their performance in auditory closure, figure-ground for verbal sounds in dichotic listening, sound source localization, and temporal processing.

Changes in figure-ground for verbal sounds with post-ACAT skill adequacy were similar to a study^(14)^ that considered the therapeutic approach a possibility to manage the auditory and cognitive deficits in individuals with mild TBI. This is also confirmed in the study by Marangoni and Gil^(15)^, although they only assessed individuals with severe TBI.

Another study^(16)^ with children and adolescents with various degrees of TBI also indicated CAP disorder with abnormal results in auditory skills, such as auditory closure, figure-ground, and temporal ordering. The authors highlighted that the main post-TBI complaints were inattention, memory difficulties, and low school performance.

The changes in assessment performance after the intervention indicate the effect of auditory training on individuals who suffered mild TBI, as previously shown in other studies^(2,17)^. These results are directly related to the ability of the central nervous system to change with auditory stimulation thanks to neural plasticity^(3,14,18)^.

Authors^(19)^ have concluded that the effects of TBI should be better understood regarding short-term and long-term communication issues in individuals with post-concussion peripheral and central auditory dysfunction.

Table 2 also shows a statistically significant difference between the ears, with better results in the right ear in the Portuguese staggered spondaic word and dichotic consonant-vowel tests. The expected right-ear advantage was also observed in the consonant-vowel test (free recall), combining with left hemisphere dominance for verbal sounds. This table demonstrates that after ACAT some procedures - such as sound localization, sequential memory test for nonverbal sounds, speech-in-white-noise, sentence identification with an ipsilateral competing message (-15), and Random Gap Detection Test - reached the expected normality, corroborating studies that assessed TBI^(15,17)^.

Other studies likewise found changes in the Portuguese staggered spondaic word test performance in individuals with mild TBI, as in the present one^(20-22)^. These researchers considered it important to investigate the injury and its relationship with cognitive factors during the recovery period and its long-term consequences. They also concluded that individuals with mild TBI may present damage to the central auditory system, indicating specific auditory rehabilitation.

Table 3 shows that most individuals reported a considerable and/or significant improvement regarding the questionnaire items, revealing they had perceived an improvement in questions involving performance in a noisy environment, in the level of attention and alertness, self-esteem, and increased attention span.

The consulted literature refers to some symptoms resulting from a concussion, such as cognitive changes, blurred vision, emotional problems such as sadness and depression, and sleep disorders. Symptoms are often invisible because there is no change in the brain structure and are difficult to detect by conventional imaging, which is why it depends heavily on each patient's report of symptoms. Authors^(23)^ suggested identifying markers of oculomotor and vestibular function to monitor mild TBI and manage the peripheral and central auditory system, which can impair listening skills.

The results of this study were consistent with behavioral CAP test results, which, as previously mentioned, were better in speech-in-noise, sound duration, temporal processing, and sequential memory for verbal and non-verbal sound tests after ACAT. This allows for inferences in the extent of post-training performance improvements in the affected auditory skills. Other studies^(5,24)^ demonstrated the effectiveness of ACAT with improved attention span.

The statistical correlation between post-ACAT self-perception questions and post-ACAT behavioral tests was analyzed (Table 4), showing a positive correlation between various tests, such as the sentence identification with an ipsilateral competing message in the left ear and sequential memory for nonverbal sounds, with improvement in following instructions and orders (Q2) - i.e., the improvement in figure-ground ability for verbal sounds in monotic listening and memory for nonverbal sounds made the individual more attentive to instructions. Regarding the dichotic consonant-vowel test, a reduction was found in the requests to repeat statements, enabling more agile and better-flowing communicative exchanges.

The Random Gap Detection Test showed that the shorter the time to perceive two sounds, the better the individual's ability to memorize orders and instructions, demonstrating that the improvement in temporal processing had an impact on the ability to memorize sequential stimuli (Table 4). This ability is reflected in everyday communication performance with one or more people, as attention in dialogue is maintained by the information processing speed during spontaneous conversation.

The duration pattern test was positively correlated with reported improvements in following orders and instructions (Q2), attention span (Q7), and talking on the phone and watching TV (Q10). Better skills in analyzing sound duration were also reported, indicating improved temporal processing, as demonstrated in the post-ACAT assessment results. This improvement indicates that individuals improved their ability to maintain focus and attention, especially to discriminate sounds, demonstrating better temporal ordering performance - i.e., the individual began to perceive the sound in less detection time when two sounds are presented, being consistent with improved phonological aspects and auditory discrimination of speech, corroborating other studies^(25,26)^ that reported improved hearing skills after the auditory training, adapting skills in individuals with severe TBI.

An author^(27)^ stated that temporal processing is one of the most important and necessary functions in the neural speech decoding process to discriminate rapid and successive sound cues over a period of time. It is also important for the development of language and reading skills in both quiet and competing noise. Therefore, the individual's ability to identify and process auditory information has an important contribution since auditory, cognitive, and language processes are directly linked to speech processing.

This skill in the present study participants impaired speech perception and, consequently, language skills. These data agree with the impairment of temporal processing skills observed in the behavioral assessment, related to temporal resolution and sound duration, and the impairment of speech recognition in noise.

Although the instrument was designed to evaluate self-perceived ACAT effectiveness, the national literature has seldom used it for this purpose. This study extracted qualitative information about the daily life situations of individuals with mild TBI regarding auditory performance, showing important improvements in the perception of cognitive behaviors.

The study findings revealed improved post-ACAT auditory behaviors of speech perception and auditory discrimination, resulting in improved communication capacity.

Mild TBI causes various long-term symptoms besides hearing difficulties, whose approach by the scientific community has been limited. Hence, attention is called to the quality of life addressed in other studies with patients who suffered brain injury. Authors^(28)^ who used a quality-of-life self-perception questionnaire in individuals with TBI observed that symptoms are likely to appear in older people, females, and those with a lower educational attainment. Thus, they recommended that patients be treated and monitored more closely, controlling the sequelae of the injury from the acute to chronic recovery.

Authors^(29)^ identified the most frequent symptoms in 400 university athletes 21 days after concussion and observed that the cognitive symptom was the most reported, followed by sleep, physical, and emotional changes.

A study^(30)^ identified that patients with mild TBI who were discharged from the hospital 1 month after the brain injury had a negative emotional response associated with a decrease in quality of life. Therefore, the authors considered it essential to identify post-concussion symptoms early and begin appropriate interventions to improve their quality of life, including CAP assessment and rehabilitation with ACAT.

Therefore, the findings demonstrated the importance of applying the self-perception questionnaire not only in research but also in clinical practice to show patients the correlation between behavioral CAP assessment results before and after ACAT - which contributes to new scientific evidence in the rehabilitation of individuals with acquired neurological injuries.

The limitation of this study includes the small sample and scarce literature to relate the findings, thus indicating the need for further studies on the topic, especially with the instrument used in this one, whether in individuals with TBI or other acquired neurological injuries.

Lastly, the study is relevant to the scientific community to emphasize that even mild TBI can compromise the auditory system and that the ACAT intervention can improve their hearing skills and everyday activities. Hence, the greatest contribution of this study is the improvement of this population’s quality of life, especially considering the predominance of young people with TBI.

## CONCLUSION

This study demonstrated that the self-perception questionnaire responses were consistent with the behavioral CAP test results, with better results after the ACAT in the speech-in-noise, sound duration, temporal processing, and sequential memory for verbal and non-verbal sounds test, resulting in fewer auditory behavior complaints, quantified with the questionnaire.
