# Supplementary material for: Central auditory processing and self-perception questionnaire after acoustically controlled auditory training in individuals with mild traumatic brain injury
Source: Codas. 2024 Apr 26;36(2):e20230048. doi: 10.1590/2317-1782/20232023048en (PMC11086975; doi:10.1590/2317-1782/20232023048en)
Supplement: Translation

# Translation: Processamento auditivo central e questionário de autopercepção pós-treinamento auditivo acusticamente controlado em indivíduos com traumatismo cranioencefálico leve

**DOI:** 10.1590/2317-1782/20232023048pt

**Authors:** Ana Karina Lima Buriti, Cyntia Barbosa Laureano Luiz, Laís Rocha de Barros Oliveira, Italo Capraro Suriano, Daniela Gil

**Affiliations:** 1 Departamento de Fonoaudiologia, Universidade Federal de São Paulo - UNIFESP - São Paulo (SP), Brasil.

**Keywords:** Audição, Percepção de Fala, Percepção Auditiva, Cognição, Lesão Cerebral, Qualidade de Vida

## Abstract

**Objetivo:**

Correlacionar os resultados da avaliação comportamental do processamento auditivo central e do questionário de autopercepção após o treinamento auditivo acusticamente controlado.

**Método:**

Foram avaliados dez indivíduos com média de idade de 44,5 anos, que sofreram traumatismo cranioencefálico de grau leve. Os indivíduos foram submetidos a avaliação comportamental do processamento auditivo central e também responderam ao questionário de autopercepção “Treinamento Auditivo Formal” após a intervenção terapêutica. O questionário foi composto por questões referentes a percepção auditiva, compreensão de ordens, solicitação de repetição de enunciados, ocorrência mal-entendidos, tempo de atenção, desempenho auditivo em ambiente ruidoso, comunicação ao telefone e autoestima e os pacientes foram solicitados a assinalar a frequência de ocorrência dos comportamentos listados.

**Resultados:**

As habilidades auditivas de figura-fundo e memória para sons em sequência e processamento temporal correlacionaram-se com melhora para seguir instruções, diminuição das solicitações de repetições e aumento do tempo de atenção e melhora da comunicação e da compreensão ao telefone e para assistir TV.

**Conclusão:**

Observou-se adequação das habilidades auditivas de fechamento auditivo, figura fundo, e processamento temporal na avaliação pós-treinamento auditivo acusticamente controlado, além de redução das queixas quanto ao comportamento auditivo.

## INTRODUÇÃO

O traumatismo cranioencefálico (TCE) é provocado por uma pancada ou abalo violento sobre o crânio, com repercussão no cérebro. Em relação à gravidade da lesão cerebral, os TCE são classificados pelo nível de consciência no momento da admissão hospitalar após o acidente. Para tal, é utilizada a Escala de Coma de Glasgow (ECG), na qual avalia-se a abertura ocular, a resposta verbal e a resposta motora do paciente, atribuindo-se um escore que varia de 3 a 15. Um TCE é considerado leve quando este escore varia de 13 a 15; TCE moderado entre 9 e 12; e o TCE grave entre 3 e 8^(1)^.

Sabe-se da importância da avaliação do processamento auditivo central em pacientes que sofreram traumatismo cranioencefálico (TCE), porém, nos traumatismos de grau leve, os sintomas, tais como redução de velocidade de processamento da informação e *déficit* de atenção, orientação, função executiva e linguagem podem surgir em longo prazo, e que consequentemente poderiam repercutir na capacidade do indivíduo em processar as informações auditivas^(2).^

Em um manuscrito americano, houve relato de transtorno de processamento auditivo central em mais de 55% dos pacientes que sofreram TCE, tanto em adultos como em crianças. Os autores recomendaram que os pacientes com TCE fossem encaminhados para abordagens terapêuticas que propiciassem a melhora das habilidades auditivas por meio do treinamento auditivo, no intuito de compensar os prejuízos residuais, utilizando recursos centrais *top-down* (do sistema nervoso para a periferia), ou seja, com estratégias metacognitivas, cognitivas e habilidades metalinguísticas^(3)^.

As recomendações internacionais citaram a importância das estratégias e exercícios do treinamento auditivo para promover eficientes percepções e minimizar déficits funcionais de escuta, por meio da plasticidade e reorganização cortical nos indivíduos com transtorno do processamento auditivo central. Destacaram ainda, a especificidade de cada indivíduo, principalmente os que têm comorbidades, a exemplo do TCE, não devendo-se super ou subestimar a capacidade cognitiva, de linguagem e intelectual, comprometendo a motivação, aspecto essencial ao processo terapêutico^(4)^.

Werff^(2)^ afirmou que é improvável que o TCE resulte em danos apenas em centros específicos da audição central. No entanto, é importante avaliar as manifestações auditivas centrais nos TCEs leves, realizando, assim, um planejamento terapêutico fonoaudiológico com a intenção de induzir as alterações neurofisiológicas e melhorar as habilidades auditivas. O autor destacou que não há uma bateria padrão de testes, mas devem ser incluídos, no mínimo, os testes de fala no ruído, de resolução temporal, de escuta dicótica, em atividades de separação binaural ou atenção seletiva.

As modificações ambientais são uma das orientações válidas para indivíduos com TCE leve, as quais possibilitam a melhora ao acesso e clareza ao som, melhora da relação sinal-ruído e aumento da capacidade de ouvir e aprender com sinais auditivos. Estas modificações podem e devem relacionar-se às estratégias de treinamento cognitivo^(2)^, utilizado por uma equipe multiprofissional, incluindo uma proposta de treinamento auditivo em cabina acústica, conhecida como treinamento auditivo acusticamente controlado (TAAC).

As diretrizes internacionais já recomendaram que profissionais da saúde ou educadores selecionassem crianças ou adultos com possíveis indicadores de risco para o transtorno do PAC, utilizando inventários ou questionários que identifiquem possíveis alterações relacionadas à compreensão auditiva, áreas acadêmicas, habilidades sociais e de local de trabalho. Porém, os questionários auxiliam os profissionais na identificação de queixas e mapeamento de dificuldades, mas não substituem a avaliação completa indicada para o diagnóstico de transtorno do processamento auditivo central (TPAC)^(5)^.

Em relação aos questionários de autopercepção muitos são indicados na literatura nacional na área da audição, mas em indivíduos com comorbidades, observou-se uma escassez de pesquisas para análise após diagnóstico e intervenção terapêutica do processamento da informação, especificamente envolvendo o TCE leve, revelando uma lacuna na literatura^(6-7)^.

Dessa forma, torna-se relevante investigar o impacto do traumatismo cranioencefálico de grau leve, na capacidade de processamento das informações auditivas e também na autopercepção do indivíduo após uma abordagem terapêutica. A contribuição deste estudo está na possibilidade de validar do ponto de vista do próprio paciente, os benefícios da abordagem terapêutica implementada.

Sendo assim, o presente estudo teve o objetivo de correlacionar os resultados da avaliação comportamental do processamento auditivo central e um questionário de autopercepção após o treinamento auditivo acusticamente controlado em indivíduos com TCE leve.

## MÉTODO

Trata-se de uma pesquisa quantitativa, descritiva, realizada no ambulatório de audiologia clínica da Disciplina dos Distúrbios da Audição do Departamento de Fonoaudiologia da Universidade Federal de São Paulo (UNIFESP) e aprovada pelo Comitê de Ética em Pesquisa da Universidade Federal de São Paulo, sob o número 1.844.535.

A amostra foi constituída por dez indivíduos, sendo dois do sexo feminino e oito do sexo masculino, com idades entre 16 e 64 anos, com diagnóstico médico de traumatismo cranioencefálico fechado leve.

A seleção e captação da amostra foram realizadas no ambulatório de Neurotrauma e Neurocirurgia do Hospital São Paulo. Os indivíduos que aceitaram participar do estudo assinaram o termo de consentimento livre esclarecido e foram convocados para uma consulta de avaliação inicial para assegurar os critérios de inclusão do estudo: ausência de queixas prévias ou atuais de afecções do sistema auditivo; ter sofrido o TCE no mínimo há 4 meses e no máximo há 12 meses; limiares auditivos menores ou iguais a 25 dBNA entre 250 e 4000Hz, bilateralmente; timpanometria com curvas tipo A, ausência de alterações do comportamento ou psiquiátricas diagnosticadas e/ou evidentes.

Em seguida, foi realizada a avaliação comportamental do processamento auditivo central, a qual foi constituída por dez testes a saber: teste de localização sonora e teste de memória para sons verbais e não verbais em sequência, fala com ruído, identificação de sentenças com mensagem competitiva ipsilateral, dicótico de dissílabos alternados, padrão de duração com tom puro^(8)^, dicótico consoante-vogal, teste de fusão auditiva randomizado e limiar diferencial de mascaramento. Foi utilizado o audiômetro da marca *Grason-Stadler*, modelo GSI-61, utilizando o fone de modelo TDH-50P.

Após essa avaliação, os indivíduos foram convidados a comparecer a 10 sessões do treinamento auditivo acusticamente controlado (TAAC), obedecendo ao protocolo proposto por Dias e Gil^(9)^, que estabelece uma sessão semanal, com duração de cinquenta minutos cada, realizadas em cabina acústica.

Durante as sessões, foram propostas atividades, organizadas em grau crescente de complexidade visando o treinamento das habilidades auditivas a serem estimuladas, apresentados por estímulos gravados em CD, por meio de fones auriculares e via audiômetro em condições de escuta dicótica, monótica ou diótica progressivamente adversas, ou seja, do nível mais fácil para o mais difícil.

No presente estudo, o programa de TAAC teve duração média de três meses para cada paciente, incluindo faltas e feriados. Vale ressaltar que as atividades propostas foram iguais para todos os participantes do estudo.

Nas sessões de TAAC foram propostas atividades envolvendo as habilidades de: reconhecimento e discriminação de sons verbais e não verbais; ordenação temporal; resolução temporal; figura fundo para sons verbais e não verbais e fechamento auditivo (Quadro 1). As tarefas e a relação sinal ruído (S/R) foram organizadas de maneira progressiva, segundo o nível de complexidade. Para passar à etapa subsequente do treinamento auditivo, o indivíduo deveria apresentar, no mínimo, 70% de acertos^(10)^.

**Quadro 1:** Cronograma de atividades do treinamento auditivo acusticamente controlado

1ª SESSÃO	Figura-fundo para frases.
2ª SESSÃO	Figura-fundo para palavras: Escuta Direcionada com Dígitos OD + Figura fundo para sons não verbais OE.
3ª SESSÃO	Figura-fundo para palavras: Escuta Direcionada com Dígitos OE + Figura fundo para sons não verbais OD.
4ª SESSÃO	Integração binaural com dígitos e sons não verbais + Fala com ruído com frases.
5ª SESSÃO	Fechamento Auditivo (Fala com ruído: frases, figuras e palavras) OD e OE.
6ª SESSÃO	Aspectos Temporais (Padrão de Intensidade) + Aspectos Temporais (Padrão de Duração: audiômetro e flauta).
7ª SESSÃO	Aspectos Temporais (Padrão de duração: tom puro).
8ª SESSÃO	Aspectos Temporais (Padrão de Frequência: audiômetro, tom puro).
9ª SESSÃO	Aspectos Temporais (Padrão de Frequência: flauta + Figura-fundo para sílabas (OD).
10ª SESSÃO	Figura-fundo para sílabas OE e Integração Binaural com sílabas.
Reavaliação	Reavaliação PAC + Questionário autopercepção.

**Legenda:** OD: orelha direita; OE: orelha esquerda; PAC: processamento auditivo central

Ao final das 10 sessões de TAAC, os indivíduos foram reavaliados utilizando o mesmo protocolo inicial. Os critérios para análise de cada teste utilizado, tanto no pré como no pós-treinamento, foram os propostos por Pereira^(11,12)^.

Em seguida, o indivíduo respondeu a um questionário de autopercepção “Questionário pós-Treinamento Auditivo Formal”, traduzido e adaptado para o português brasileiro por Dias e Gil^(9)^, utilizado em indivíduos com e sem perda auditiva, visando pontuar formalmente as modificações provocadas pelo treinamento auditivo, do ponto de vista do próprio indivíduo ou de sua família. Esse questionário é composto por 12 perguntas relacionadas à percepção de melhora auditiva, compreensão de ordens, evolução acadêmica, solicitação de repetição de enunciados, redução de mal-entendidos, aumento do tempo de atenção, desempenho auditivo em ambiente ruidoso, melhora ao falar no telefone e assistir televisão e autoestima. O indivíduo foi orientado a atribuir, para cada item, uma resposta de autopercepção pautada em uma escala, que variou entre zero e quatro, sendo: 0-nenhuma melhora; 1-melhora sutil, porém importante; 2-melhora moderada; 3-melhora considerável e 4-melhora significativa (Quadro 2). Para um melhor entendimento dessa escala, a pesquisadora deu o exemplo em percentual, variando de 0 a 100% de melhora, assim, o paciente conseguiu responder com clareza a cada questão.

**Quadro 2:** Questionário de autopercepção (após treinamento auditivo acusticamente controlado)

**Análise das respostas:** 0**-** nenhuma melhora; 1- melhora sutil, porém importante; 2- melhora moderada; 3- melhora considerável; 4- melhora significativa.	0	1	2	3	4
Q1. Você observa melhora na audição?					
Q2. Foi observada melhora para seguir instruções, ordens...					
Q3. A comunicação tem sido mais fácil?					
Q4. Houve alguma melhora acadêmica (leitura, soletração?)					
Q5. Houve redução na solicitação de repetição de enunciados?					
Q6. Os mal-entendidos na comunicação diminuíram?					
Q7. O tempo de atenção aumentou?					
Q8. O desempenho auditivo em ambiente ruidoso melhorou?					
Q9. Houve melhora no nível de atenção e alerta?					
Q10. Houve melhora ao falar no telefone, assistir TV, ouvir rádio, etc ?					
Q11. Houve melhora quanto a autoestima?					
Q12. Descreve outras mudanças observadas durante ou após o período de treinamento auditivo formal.					

Os dados foram analisados descritivamente com base nas frequências absolutas e percentuais para as variáveis discretas e das medidas de média, desvio padrão (média ± DP) e mediana para as variáveis numéricas. Utilizou-se o teste t-Student pareado e o teste Wilcoxon para análise estatística inferencial. A margem de erro utilizada na decisão dos testes estatísticos foi de p< 0,05%.

## RESULTADOS

A caracterização da amostra segundo a idade variou de 16 a 64 anos, com média de 44,50 anos, desvio padrão de 18,32 anos e mediana de 53,00 anos. O sexo masculino foi representado pela maioria dos pacientes analisados (80%). Foram observadas diversas causas para o TCE, a saber, queda de altura maior que dois metros (40%), queda da própria altura (30%) e acidente automobilístico (30%).

A lesão cerebral dos indivíduos do presente estudo acometeu o lado esquerdo (40%), seguida de lesão bilateral (40%) e em apenas 20% atingiu o lado direito.

Na Tabela 1, segue a caracterização da amostra selecionada, de acordo com idade, sexo, diagnóstico médico da lesão primária ou secundária ao TCE de grau leve, com Glascow 13 a 15 no momento da admissão ao hospital, o lado da lesão, se foi submetido a procedimento cirúrgico e uso de medicações.

**Tabela 1:** Caracterização da amostra

**N**	**Idade**	**Sexo**	**Escolaridade**	**Diagnóstico médico**	**Lado lesão**	**Cirurgia**	**Internação**	**Medicação**
1	51	M	Ensino médio incompleto	Hematoma subdural crônico	Esquerdo	Sim	3 dias	Não
2	64	M	Ensino médio completo	Hematoma Intraparenquimatoso frontal e parietal	Esquerdo	Não	3 dias	Sertralina
3	58	M	Ensino Fundamental incompleto	Hematoma subdural agudo	Esquerdo	Não	4 dias	Não
4	32	F	Fundamental incompleto	Contusão frontal mediana + Contusão temporal	Bilateral	Não	1 dia	Não
5	16	M	Ensino médio incompleto	Empiema extradural frontal	Esquerdo	Sim	2 meses - 3 UTI.	Não
6	64	F	Superior completo	Contusão frontal	Direito	Não	3 dias	Sertralina
7	28	M	Ensino médio completo	Hematoma extradural parietal	Direito	Sim	4 dias	Não
8	21	M	Ensino médio completo	Concussão difusa	Bilateral	Não	Não	Fluoxetina
9	55	M	Ensino fundamental incompleto	Hematoma extradural temporal + Hematoma epidural agudo + Hematoma Subdural agudo laminar fronto temporal	Bilateral	Sim	5 dias	Não
10	56	M	Ensino médio completo	Hematoma Subdural crônico	Bilateral	Sim	3 dias	Não

**Legenda:** M: masculino; F: feminino; UTI: unidade de terapia intensiva

Na Tabela 2 estão apresentados os resultados dos testes comportamentais do processamento auditivo central (PAC) pré e pós-TAAC.

**Tabela 2:** Avaliação comportamental do processamento auditivo central (PAC) pré e pós-TAAC, considerando os testes utilizados e lado da orelha (n=10)

		**Avaliação**		
**Testes comportamentais**	**Orelha**	**Pré**	**Pós**	**Valor de p**
		Média ± DP (Mediana)	Média ± DP (Mediana)	
				
**TFRB** (%)	Direita	67,60 ± 16,91 (70,00)	76,80 ± 10,29 (80,00)	p^(1)^ = 0,127
	Esquerda	65,60 ± 7,11 (64,00)	74,40 ± 10,36 (74,00)	**p^(2)^** = 0,042*
	**Valor de p**	p^(1)^ = 0,678	p^(1)^ = 0,329	
				
**SSI (S=/R-15)** (%)	Direita	63,00 ± 17,67 (65,00)	75,00 ± 15,09 (70,00)	p^(1)^ = 0,074
	Esquerda	61,00 ± 17,29 (70,00)	71,00 ± 18,53 (80,00)	p^(2)^ = 0,093
	**Valor de p**	p^(1)^ = 0,678	p^(1)^ = 0,399	
				
**SSW** (%)	Direita	83,00 ± 17,11 (88,75)	89,75 ± 12,44 (93,75)	p^(2)^ = 0,016*
	Esquerda	81,75 ± 13,02 (85,00)	85,00 ± 16,41 (90,00)	p^(2)^ = 0,108
	**Valor de p**	p^(2)^ = 0,435	p^(1)^ = 0,038*	
				
**TDCV** (acertos)	Direita	10,40 ± 3,44 (10,50)	12,10 ± 2,96 (11,50)	p^(1)^ = 0,169
	Esquerda	8,00 ± 3,20 (7,50)	6,50 ± 2,55 (6,50)	p^(1)^ = 0,169
	**Valor de p**	p^(1)^ = 0,217	p^(1)^ = 0,009*	
				
**TDCV** (erros)		5,60 ± 3,37 (4,00)	5,20 ± 1,40 (5,00)	p^(1)^ = 0,674
				
**TLS** (%)		68,00 ± 19,32 (80,00)	82,00 ± 19,89 (80,00)	p^(2)^ = 0,020*
				
**TMSV** (%)		50,00 ± 36,00 (50,00)	73,33 ± 21,08 (66,66)	p^(^1^)^ = 0,089
				
**TMSNV** (%)		63,33 ± 36,68 (66,66)	73,33 ± 34,43 (83,33)	p^(^2^)^ = 0,257
				
**RGDT** (ms)		10,25 ± 6,67 (8,13)	5,60 ± 2,77 (4,50)	p^(1)^ = 0,036*
				
**TPD** (%)		62,53 ± 26,85 (64,97)	79,32 ± 24,40 (88,33)	p^(1)^ = 0,133
				
**MLD** (dB)		12,80 ± 3,43 (12,00)	12,20 ± 2,57 (12,00)	p^(1)^ = 0,691
				

* Diferença significativa ao nível de 5,0%

^(1)^Através do teste t-Student pareado;

^(2)^Através do teste Wilcoxon pareado

**Legenda:** TFRB: teste de fala com ruído branco; SSI (MCI): identificação de sentenças sintéticas (mensagem competitiva ipsilateral); SSW: teste dicótico de dissílabos alternados; TDCV: teste dicótico consoante vogal; TLS: teste de localização sonora; TMSV: teste de memória sequencial verbal; TMSNV: teste de memória sequencial de sons não-verbais; TPD: teste padrão de duração; RGDT: *Random Gap Detection Test*; MLD: Limiar Diferencial de mascaramento

Observou-se diferença estatisticamente significante nos testes de fala no ruído à orelha esquerda (p=0,042), no SSW à orelha direita (p=0,016), no TLS (p=0,020) e no RGDT (p=0,036) comparando as avaliações pré e pós, com melhor desempenho na reavaliação, ou seja, após o treinamento auditivo acusticamente controlado. Foi observada também, diferença estatisticamente significante entre as orelhas direita e esquerda na avaliação pós, para os testes SSW (p=0,038) e TDCV (p=0,009) (Tabela 2).

Em relação à dominância hemisférica, observou-se no TDCV a vantagem da orelha direita nas avaliações pré e pós.

Na Tabela 3 estão apresentadas as respostas dos indivíduos em relação à autopercepção dos comportamentos do processamento da informação pós-TAAC.

**Tabela 3:** Distribuição de frequências das respostas do questionário TAF

	**Níveis de respostas quanto a melhora**									
**TAF**	**Nenhuma**		**Sutil**		**Moderada**		**Considerável**		**Significativa**	
	n	%^(^1^)^	n	%^(1)^	n	%^(1)^	n	%^(1)^	n	%^(1)^
										
**Q1**	-	-	-	-	1	10,0	5	50,0	4	40,0
										
**Q2**	1	10,0	-	-	2	20,0	4	40,0	3	30,0
										
**Q3**	1	10,0	1	10,0	1	10,0	4	40,0	3	30,0
										
**Q4**	2	20,0	2	20,0	2	20,0	1	10,0	3	30,0
										
**Q5**	-	-	1	10,0	3	30,0	3	30,0	3	30,0
										
**Q6**	1	10,0	-	-	2	20,0	4	40,0	3	30,0
										
**Q7**	-	-	1	10,0	1	10,0	3	30,0	5	50,0
										
**Q8**	2	20,0	-	-	1	10,0	1	10,0	6	60,0
										
**Q9**	1	10,0	-	-	2	20,0	1	10,0	6	60,0
										
**Q10**	1	10,0	1	10,0	-	-	5	50,0	3	30,0
										
**Q11**	1	10,0	1	10,0	-	-	2	20,0	6	60,0
										

^(1)^Os valores percentuais foram obtidos do número total de 10 pacientes analisados

**Legenda:** TAF: treinamento auditivo formal; Q: questão; Q1: observa melhora na audição?; Q2: observada melhora para seguir instruções, ordens?; Q3: comunicação mais fácil?; Q4: melhora acadêmica, leitura, soletração?; Q5: redução na solicitação de repetição de enunciados?; Q6: mal-entendidos na comunicação diminuíram?; Q7: tempo de atenção?; Q8: desempenho auditivo em ambiente ruidoso; Q9: melhora no nível de atenção e alerta?; Q10: melhora ao falar no telefone, assistir TV, ouvir rádio; Q11: melhora na autoestima?

Na Tabela 3, observou-se maior concentração de respostas nas colunas de melhora considerável e significante, sendo destacadas na coluna de significante, as questões Q8 (desempenho auditivo em ambiente ruidoso), Q9 (nível de atenção e alerta?) e Q11 (quanto a autoestima?). Para a coluna considerável, foram destacadas as questões de Q1 (melhora na audição) e Q10 (ao falar no telefone, assistir TV, ouvir rádio), seguida das questões Q2, Q3 e Q6.

A Tabela 4 apresenta os resultados estatisticamente significantes, das respostas do questionário TAF correlacionado aos resultados dos testes comportamentais da informação pós-TAAC.

**Tabela 4:** Correlação do questionário de treinamento auditivo formal com os testes comportamentais pós-Treinamento auditivo acusticamente controlado

	**TAF**					
**PAC - Pós**	**Q2**	**Q5**	**Q7**	**Q8**	**Q9**	**Q10**
**TFRB**						
Direita	-	-	-	-	-0,763 (0,010)^(2)*^	-
**SSI**						
Esquerda	0,911 (< 0,001)^(2)*^	-	-	-	-	-
**TDCV**						
Direita	-	0,734 (0,016)^(^1)^*^	-	-	-	-
**TMSV**	-	-	-	-0,639 (0,047)^(2)*^	-	-
**TMSNV**	0,633 (0,050)^(^2)^*^	-	-	-	-	-
**RGDT**	-0,823 (0,003)^(2)*^	-	-	-	-	-
**TPD**	0,779 (0,008)^(2)^*	-	0,710 (0,021)^(2)*^	-	-	0,684 (0,029)^(2)*^

* Estatisticamente diferente de zero

^(1)^Correlação de Pearson;

^(2)^Correlação Spearman

**Legenda:** PAC: processamento auditivo central; TAF: treinamento auditivo formal; Q2: observada melhora para seguir instruções, ordens?; Q5: redução na solicitação de repetição de enunciados?; Q7: tempo de atenção?; Q8: desempenho auditivo em ambiente ruidoso; Q9: melhora no nível de atenção e alerta?; Q10: melhora ao falar no telefone, assistir TV, ouvir rádio; TFRB: teste de fala com ruído branco; SSI (MCI): identificação de sentenças sintéticas (mensagem competitiva ipsilateral); TDCV: teste dicótico consoante vogal; TMSV: teste de memória sequencial verbal; TMSNV: teste de memória seqüencial de sons não-verbais; RGDT: *Random Gap Detection Test*; TPD: teste padrão de duração.

Quanto aos testes comportamentais pós-TAAC correlacionados ao questionário aplicado após o programa de treinamento, os pacientes que referiram melhora para seguir instruções, ordens (Q2) também apresentaram melhores resultados nos testes SSI (p< 0,001) à orelha esquerda, TMSNV (0,050), RGDT (0,003) e o TPD (p=0,008), uma vez que as correlações foram positivas.

Os indivíduos referiram redução na solicitação de repetição de enunciados (Q5) com significância para o teste dicótico consoante vogal com vantagem à direita (p=0,016). Em relação às dificuldades de atenção houve aumento no tempo de atenção (Q7) e melhoras em falar ao telefone, assistir TV, ouvir rádio (Q10) referido pelos pacientes com diferença estatisticamente significante apenas no TPD (p=0,021) e (p=0,029), respectivamente (Tabela 4). Portanto, essa correlação foi positiva, podendo ser afirmado que quanto maior a percepção da melhora do tempo de atenção, melhor o desempenho nos aspectos temporais envolvidos na identificação da duração dos sons. Pode-se inferir que ao ser capaz de se manter mais atento o indivíduo melhorou sua capacidade de diferenciar os sons quanto à duração, cujo impacto nas trocas comunicativas reside no melhor aproveitamento do conteúdo supra segmentar da fala (tonicidade, entonação).

Em relação ao desempenho auditivo em ambiente ruidoso (Q8) e nível de atenção e alerta (Q9), foi observada diferença estatística para o teste TMSV (p=0,047) e TFRB (p=0,010), respectivamente. Essa correlação foi negativa, e portanto, pode-se dizer que quanto pior o desempenho auditivo em ambiente ruidoso, melhor o desempenho para memorizar sons verbais. Observou-se também, que quanto pior o desempenho auditivo em ambiente ruidoso, melhor será o nível de atenção e alerta.

## DISCUSSÃO

O traumatismo cranioencefálico de grau leve provoca um aumento da difusão na substância cinzenta da região cortical, e após quatro meses da lesão diminuem os sinais neurocomportamentais e fisiológicos. Autores^(13)^ referiram que o indivíduo pode não apresentar alterações na região cortical ou subcortical, no entanto, perda neuronal em pacientes com TCE leve poderá surgir mais tardiamente após lesão. Nesse sentido, ressalta-se que os sintomas auditivos podem surgir nessa população, sendo importante realizar a avaliação audiológica periférica e central após os quatro meses de lesão cerebral, período de estabilidade de tantos outros sintomas clínicos.

Na análise descritiva da avaliação comportamental do processamento auditivo central entre as avaliações pré e pós-TAAC (Tabela 2), foi observada diferença estatisticamente significante na comparação das médias de acertos pré e pós-TAAC nos testes de fala no ruído à orelha esquerda, SSW à orelha direita e teste de localização sonora e RGDT, demonstrando que o treinamento auditivo proporcionou melhor desempenho nas habilidades de fechamento auditivo, figura-fundo para sons verbais em escuta dicótica, localização da fonte sonora e processamento temporal.

As alterações na habilidade de figura fundo para sons verbais com adequação da habilidade após o TAAC mostrou-se semelhante a um estudo^(14)^, que considerou a proposta terapêutica como uma possibilidade de gerenciar as ocorrências de *déficits* auditivos e cognitivos em indivíduos com TCE leve. Isso também se confirma com o estudo de Marangoni e Gil^(15)^, apesar de terem avaliado indivíduos com TCE grave.

Outro estudo^(16)^ que envolveu crianças e adolescentes com TCE em graus variados, também indicou TPAC com resultados anormais nas habilidades auditivas, tais como fechamento auditivo, figura-fundo e ordenação temporal. Os autores destacaram as principais queixas pós-TCE que foram desatenção, dificuldades de memória e baixo rendimento escolar.

A mudança do desempenho nos testes da avaliação pós-intervenção reflete o efeito do treinamento auditivo nos indivíduos que sofreram TCE leve, como já mostrado em outros estudos^(2,17)^. Esses resultados estão diretamente relacionados à capacidade do sistema nervoso central de mudar frente à estimulação auditiva, propiciada pela plasticidade neural^(3,14,18)^.

Autores^(19)^ concluíram que os efeitos do TCE deveriam ser melhor compreendidos em relação às questões da comunicação de curto e longo prazos em indivíduos com disfunção auditiva periférica e central pós-concussão.

Na comparação entre as orelhas, ainda na Tabela 2, observou-se diferença estatisticamente significante a favor da orelha direita nos testes SSW em Português e Dicótico Consoante-Vogal. Observou-se também a esperada vantagem da orelha direita no teste consoante vogal (atenção livre), combinando com dominância hemisférica esquerda para sons verbais. Pela tabela 2, verifica-se que após o TAAC alguns procedimentos atingiram a normalidade esperada, tais como LS, TMSNV, FRB, SSI (-15) e RGDT, corroborando com estudos que avaliaram o TCE^(15,17)^.

As alterações encontradas no desempenho do teste SSW em português nos indivíduos com TCE leve do presente estudo também foram encontradas em outros estudos^(20-22)^. Estes pesquisadores consideraram importante a investigação da lesão e sua relação com os fatores cognitivos no período de sua recuperação e suas consequências em longo prazo, como também concluíram que o indivíduo com TCE leve pode apresentar danos ao sistema auditivo central, sendo indicada a reabilitação auditiva específica.

Na Tabela 3, observou-se que a maioria dos indivíduos respondeu ter percebido melhora classificada como considerável e/ou significativa em relação aos itens do questionário, revelando percepção de melhora nas questões envolvendo desempenho em ambiente ruidoso, no nível de atenção e alerta e na autoestima, além do aumento no tempo de atenção.

Na literatura consultada há referência de alguns sintomas decorrentes de uma concussão, tais como alterações cognitivas, visão embaraçada, problemas emocionais como tristeza e depressão, distúrbio do sono. Os sintomas são muitas vezes invisíveis porque não há alteração na estrutura cerebral e são difíceis de serem detectáveis por imagens convencionais, por isso que depende fortemente do relato dos sintomas de cada paciente. Autores^(23)^ sugeriram identificar marcadores da função oculomotora e vestibular para monitorar o TCE leve, além de gerenciar o sistema auditivo periférico e central, que pode prejudicar a habilidade de escuta.

Os resultados desse estudo demonstraram uma coerência com os resultados dos testes comportamentais do PAC, os quais como já apontados apresentaram melhores resultados nos testes de fala no ruído, duração dos sons e processamento temporal, memória sequencial para sons verbais e não verbais, após o TAAC. Com isso, pode-se inferir sobre o quanto o indivíduo melhorou seu desempenho nas habilidades auditivas alteradas e treinadas no programa terapêutico. Outros estudos^(5,24)^ demonstraram a eficácia do TAAC pela melhora no tempo de atenção.

Foi realizada uma análise estatística de correlação entre as questões do questionário de autopercepção (pós-TAAC) e os testes comportamentais após o TAAC (Tabela 4), observando-se correlação positiva entre diversos testes, como no teste SSI à orelha esquerda e teste de memória sequencial para sons não verbais com a melhora para seguir instruções e ordens (Q2), ou seja, a melhora da habilidade de figura-fundo para sons verbais em escuta monótica e memória para sons não verbais tornou o indivíduo mais atento às instruções. Em relação ao teste dicótico consoante-vogal, observou-se redução de solicitações de repetição dos enunciados, possibilitando maior agilidade e fluidez nas trocas comunicativas.

Em relação ao teste RGDT, foi possível observar que quanto menor o intervalo de tempo para perceber dois sons, melhor foi a capacidade do indivíduo em memorizar ordens e instruções, demonstrando que a melhora no processamento temporal repercutiu na capacidade para memorizar estímulos em sequência (Tabela 4). Essa habilidade reflete no desempenho da comunicação com uma ou mais pessoas no cotidiano, pois a atenção no diálogo se mantém pela rapidez em processar a informação durante a conversa espontânea.

Já para o teste de padrão de duração, houve correlação positiva quando o indivíduo referiu melhora para seguir ordens e instruções (Q2), melhora no tempo de atenção (Q7) e melhora ao falar ao telefone e assistir à TV (Q10). Também apresentou melhor capacidade para analisar os sons quanto à sua duração, indicando melhora de processamento temporal, conforme demonstrado nos resultados da avaliação pós-TAAC. Essa melhora indica que o indivíduo aprimorou sua capacidade de manter o foco e a atenção, principalmente sua capacidade de discriminação de sons, sendo possível verificar melhor desempenho na habilidade de ordenação temporal, ou seja, o indivíduo passou a perceber o som em menor tempo de detecção, na presença de dois sons, sendo condizentes com a melhora nos aspectos fonológicos e de discriminação auditiva da fala, corroborando com estudos^(25,26)^, segundo os quais as habilidades auditivas melhoram após o treinamento auditivo, adequando as habilidades em indivíduos com TCE grave.

Autor^(27)^ afirmou que durante o processo de decodificação neural da fala, o processamento temporal é considerado uma das funções mais importantes e necessárias para a discriminação de pistas sonoras rápidas e sucessivas em um período de tempo. Além de ser importante para o desenvolvimento de habilidades linguísticas e de leitura, no silêncio e no ruído competitivo. Portanto, a capacidade do indivíduo identificar e processar informações auditivas tem uma contribuição importante, uma vez que os processos auditivos, cognitivos e de linguagem, encontram-se diretamente ligados ao processamento de fala.

Nos indivíduos do presente estudo, essa habilidade prejudicou a percepção de fala e, consequentemente, as habilidades de linguagem. Estes dados coincidem com o prejuízo das habilidades de processamento temporal observadas na avaliação comportamental, relacionado aos aspectos de resolução temporal e de duração dos sons, bem como o prejuízo ao reconhecimento da fala na presença do ruído.

Apesar de o instrumento ter sido elaborado para avaliar a eficácia do treinamento auditivo acusticamente controlado do ponto de vista da autopercepção, foi pouco utilizado pela literatura nacional para este fim. No presente estudo, o mesmo foi capaz de extrair informações qualitativas sobre as situações da vida diária destes indivíduos com TCE leve relacionadas ao desempenho auditivo, mostrando melhoras importantes na percepção dos comportamentos cognitivos.

Os achados encontrados no presente estudo revelaram melhora nos comportamentos auditivos da percepção de fala e discriminação auditiva pós-treinamento auditivo acusticamente controlado (TAAC), refletindo em melhora da capacidade de comunicação.

Sabendo que o TCE leve provoca diversos sintomas em longo prazo, não apenas as dificuldades auditivas, e por ser uma limitação da comunidade científica em abordar esse assunto, destacam-se para a qualidade de vida que outros estudos abordam na população que sofreu lesão cerebral. Autores^(28)^ que utilizaram a ferramenta de um questionário de autopercepção da qualidade de vida em indivíduos com TCE, observaram que há chances dos sintomas surgirem em pessoas mais velhas, do sexo feminino e com menos educação básica. Portanto, recomendaram que os pacientes devessem ser monitorados e tratados mais de perto, controlando as sequelas da lesão, desde o período da recuperação aguda até a crônica.

Autores^(29)^ identificaram os sintomas mais frequentes em 400 atletas universitários 21 dias após a concussão, e observaram que o sintoma cognitivo foi o mais relatado, seguido por alterações do sono, físico e emocionais.

Estudo^(30)^ identificou que pacientes com TCE leve que deixaram o hospital um mês após a lesão cerebral, apresentaram resposta emocional negativa associada à diminuição na qualidade de vida. Com base nisso, os autores consideraram essencial identificar precocemente os sintomas pós-concussão, bem como iniciar intervenções apropriadas para melhorar a qualidade de vida dos pacientes, dentre as quais pode citar a realização da avaliação do processamento auditivo central e sua reabilitação por meio do treinamento acusticamente controlado.

Sendo assim, os achados demonstraram a importância em realizar a aplicação do questionário de autopercepção não apenas em pesquisas, mas como aplicação clínica para demonstrar ao indivíduo a correlação dos resultados da avaliação comportamental do processamento auditivo central pré e pós-TAAC, a fim de contribuir com novas evidências científicas na reabilitação em indivíduos com lesões neurológicas adquiridas.

Como limitação deste estudo, podemos citar a amostra pequena e literatura reduzida para relacionar os achados, indicando assim, a necessidade de novos estudos sobre o tema e sobretudo com o instrumento aqui apresentado, seja em indivíduos com TCE ou com outras lesões neurológicas adquiridas.

Por fim, o estudo tornou-se relevante à comunidade científica para enfatizar que o TCE apesar de leve pode comprometer o sistema auditivo e que a intervenção proposta pelo TAAC pode trazer melhora das habilidades auditivas, refletindo em suas atividades de vida diária, sendo a melhora da qualidade de vida para essa população a maior contribuição deste estudo, especialmente considerando a predominância de jovens na ocorrência dos TCEs.

## CONCLUSÃO

O presente estudo demonstrou uma coerência das respostas ao questionário de autopercepção com os resultados dos testes comportamentais do PAC, nos quais foi percebido melhores resultados após o TAAC, nos testes de fala no ruído, duração dos sons e processamento temporal, memória sequencial para sons verbais e não verbais, refletindo em redução das queixas quanto ao comportamento auditivo quantificadas pelo questionário.
